# Supplementary material for: Modified Cu-Sn Catalysts Enhance CO2RR Towards Syngas Generation
Source: Materials (Basel). 2025 Aug 30;18(17):4070. doi: 10.3390/ma18174070 (PMC12429331; doi:10.3390/ma18174070)
Supplement: Supplementary file 1 [file materials-18-04070-s001.zip › materials-3803059-supplementary (author comments).pdf]

Supporting information for

Modified Cu-Sn catalysts enhance CO2RR towards syngas generation

Daniel Herranz[a], Antonio Maroto[a], Martina Rodriguez[a], Juan Ramón Avilés Moreno[*a], Pilar Ocón[a].

a. Departamento de Química Física Aplicada, Universidad Autónoma de Madrid, C/ Francisco Tomás y Valiente 7, 28049, Madrid, Spain.

Corresponding author: juan.aviles@uam.es (J.R. Avilés Moreno)

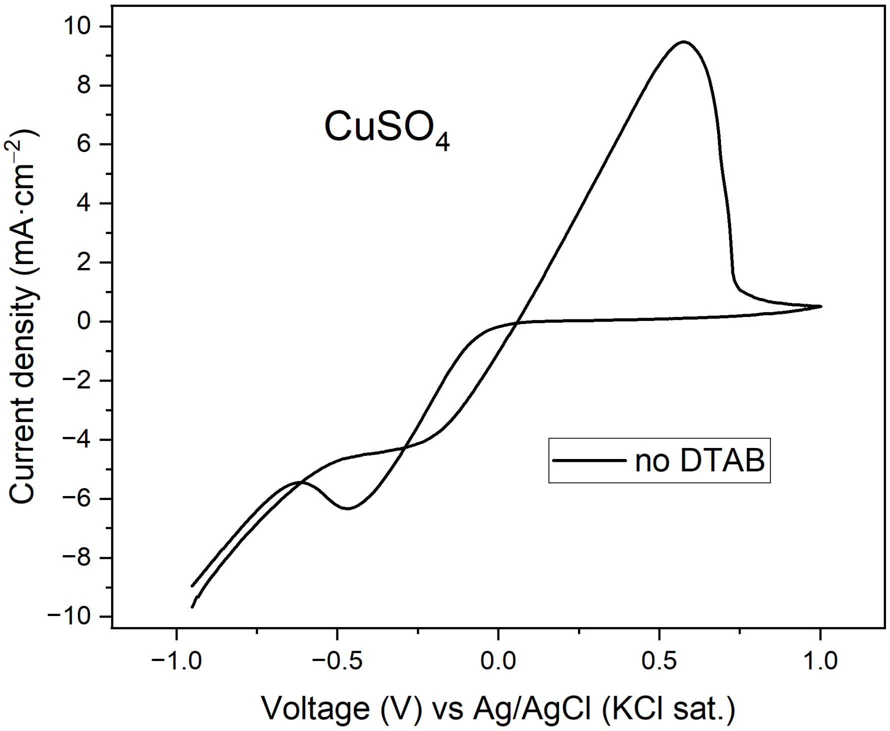

Figure S1. CV at 20 mV·s^{-1} of carbon cloth in 50 mM $CuSO_4$ at pH 2.

Table S1. CO2RR of $CuSO_4$ and $CuCl_2$ catalysts.

Catalysts	Flow ($mL \cdot min^{-1}$)	Current ($mA \cdot cm^{-2}$)	Voltage (V)	$\%CO_2$ outlet	FE to products			
					$\%H_2$	$\%CH_4$	$\%C_2H_4$	%CO
$Cu(SO_4)$	80	−25	−2.00	38.8091	93.6	0.5	1.2	4.7
$Cu(SO_4)$	20	−25	−2.29	32.4	72.8	6.3	12.8	8.0
$Cu(SO_4)$	5	−25	−2.27	27.2	93.4	1.0	2.3	3.3
$Cu(SO_4)$	3	−25	−2.29	21.9	93.3	1.5	2.1	3.1
$Cu(SO_4)$	80	−50	−2.33	27.6	65.1	10.6	15.5	8.9
$Cu(SO_4)$	20	−50	−2.58	22.4	49.8	18.7	29.0	2.4
$Cu(SO_4)$	5	−50	−2.80	17.0	54.8	26.7	17.4	1.1
$Cu(SO_4)$	3	−50	−2.85	15.3	59.6	26.5	13.0	1.0
$Cu(Cl_2)$	80	−25	−2.05	38.3	69.4	0.4	3.0	27.2
$Cu(Cl_2)$	40	−25	−2.11	34.8	67.3	0.2	5.9	26.6
$Cu(Cl_2)$	20	−25	−2.20	29.5	60.0	0.3	18.2	21.4
$Cu(Cl_2)$	5	−25	−2.29	28.7	71.7	1.1	10.0	17.2
$Cu(Cl_2)$	3	−25	−2.31	21.2	72.2	2.1	11.2	14.5
$Cu(Cl_2)$	80	−50	−2.19	39.4	60.4	0.5	28.1	11.1
$Cu(Cl_2)$	20	−50	−2.31	28.5	49.3	0.5	47.1	3.2
$Cu(Cl_2)$	5	−50	−2.38	23.9	64.3	1.3	32.3	2.1
$Cu(Cl_2)$	3	−50	−2.40	19.9	66.6	2.1	29.1	2.2

Table S2. CO2RR of Cu-Sn(2) and Cu-Sn(4.5) catalysts.

Catalysts	Flow (mL·min^{-1})	Current (mA·cm^{-2})	Voltage (V)	%CO_2 outlet	FE to products			
					%H_2	%CH_4	%C_2H_4	%CO
Cu-Sn(2)	20	−25	−2.25	41.6	87.6	0.2	0.6	11.5
Cu-Sn(2)	40	−25	−2.15	48.8	91.1	0.3	0.7	8.0
Cu-Sn(2)	80	−25	−2.05	46.9	93.8	0.2	0.6	5.5
Cu-Sn(2)	120	−25	−2.00	48.7	95.5	0.2	0.6	3.7
Cu-Sn(2)	20	−50	−2.65	36.9	91.9	0.5	0.4	7.2
Cu-Sn(2)	40	−50	−2.40	36.9	80.8	0.3	0.6	18.3
Cu-Sn(2)	80	−50	−2.30	38.5	85.2	0.2	0.4	14.2
Cu-Sn(2)	120	−50	−2.25	38.9	84.3	0.1	0.4	15.2
Cu-Sn(4.5)	20	−25	−2.52	33.8	70.4	0.4	1.1	28.1
Cu-Sn(4.5)	40	−25	−2.20	48.1	64.0	0.4	1.3	34.4
Cu-Sn(4.5)	80	−25	−2.10	51.7	60.3	0.4	1.3	38.1
Cu-Sn(4.5)	120	−25	−2.07	53.4	55.3	0.4	1.2	43.0
Cu-Sn(4.5)	20	−50	−4.44	33.2	77.2	8.6	1.8	12.3
Cu-Sn(4.5)	40	−50	−3.38	38.9	54.2	9.2	3.1	33.5
Cu-Sn(4.5)	80	−50	−2.68	44.1	49.6	1.7	2.2	46.5
Cu-Sn(4.5)	120	−50	−2.46	45.9	47.5	0.6	1.7	50.2

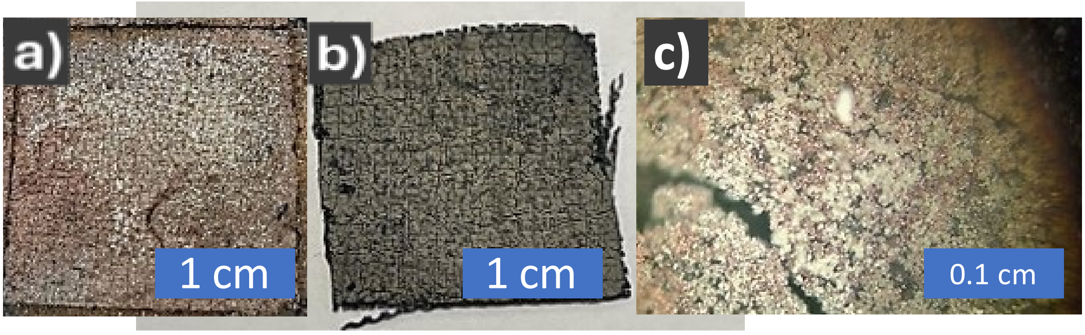

Figure S2. Pictures of a) Cu-Sn(2) and b) Cu-Sn(4.5) catalysts before CO2RR; c) is a closer picture of catalyst Cu-Sn(4.5) made by optical microscope.

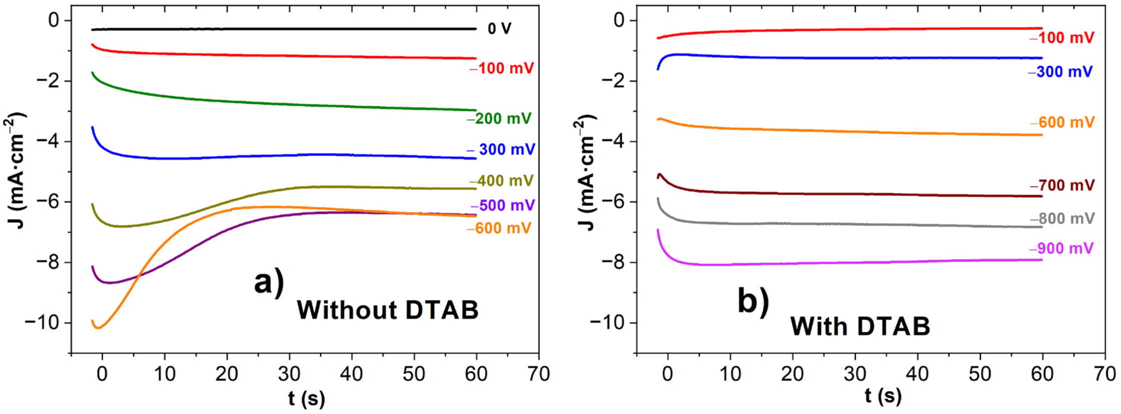

Figure S3. CAs at different voltage values vs Ag/AgCl (KCl sat.) of carbon cloth in a pH 2 solution of 50 mM $CuSO_4$ solution, a) without and b) with 5 mM DTAB.

Table S3. CO2RR performance of catalysts prepared by CA: Cu-Sn(A), Cu-Sn(B), and Cu-Sn(C).

Catalysts	Flow ($mL·min^{-1}$)	Current ($mA·cm^{-2}$)	Voltage (V)	$\%CO_2$ outlet	FE to products $\%H_2$	$\%CH_4$	$\%C_2H_4$	%CO
Cu-Sn(A)	20	−25	−2.04	37	74.1	0.0	4.6	21.3
Cu-Sn(A)	40	−25	−1.97	43	62.5	0.0	2.9	34.5
Cu-Sn(A)	80	−25	−1.88	46	57.8	0.0	1.2	41.0
Cu-Sn(A)	120	−25	−1.85	46	56.6	0.0	0.0	43.4
Cu-Sn(A)	20	−50	−2.22	36	69.6	0.0	21.2	9.1
Cu-Sn(A)	40	−50	−2.18	38	62.5	0.0	25.5	12.0
Cu-Sn(A)	80	−50	−2.14	45	57.7	0.0	15.2	27.1
Cu-Sn(A)	120	−50	−2.07	44	52.9	0.0	21.2	25.8
Cu-Sn(A)	20	−100	−2.80	29	76.1	0.0	19.2	4.7
Cu-Sn(A)	40	−100	−2.77	32	58.2	7.8	26.7	7.3
Cu-Sn(A)	80	−100	−2.66	35	49.4	3.3	35.2	12.1
Cu-Sn(A)	120	−100	−2.55	40	48.1	1.8	30.0	20.1
Cu-Sn(B)	20	−25	−2.04	57	73.6	0.0	0.0	26.4
Cu-Sn(B)	40	−25	−2.04	55	65.9	0.0	0.0	34.1
Cu-Sn(B)	80	−25	−2.01	56	64.0	0.0	0.0	36.0
Cu-Sn(B)	120	−25	−1.98	49	61.2	0.0	0.0	38.8
Cu-Sn(B)	20	−50	−2.88	44	74.0	9.6	0.0	16.3
Cu-Sn(B)	40	−50	−2.38	46	57.9	0.0	0.0	42.1
Cu-Sn(B)	80	−50	−2.23	48	51.9	0.0	0.0	48.1
Cu-Sn(B)	120	−50	−2.17	47	50.0	0.0	0.0	50.0
Cu-Sn(B)	20	−100	−3.10	18	89.3	3.9	0.6	6.3
Cu-Sn(B)	40	−100	−3.05	24	77.3	9.2	1.7	11.8
Cu-Sn(B)	80	−100	−2.74	26	59.2	16.3	7.3	17.1
Cu-Sn(B)	120	−100	−2.56	27	62.1	6.8	7.0	24.2
Cu-Sn(C)	20	−25	−2.06	41	92.3	0.0	0.0	7.7
Cu-Sn(C)	40	−25	−2.02	50	89.1	0.0	0.0	10.9
Cu-Sn(C)	80	−25	−1.99	47	65.0	0.0	0.0	35.0
Cu-Sn(C)	120	−25	−1.95	51	59.1	0.0	0.0	40.9
Cu-Sn(C)	20	−50	−2.55	19	88.9	2.0	0.6	8.5
Cu-Sn(C)	40	−50	−2.35	35	77.2	0.8	0.6	21.5
Cu-Sn(C)	80	−50	−2.20	39	62.3	0.0	1.8	36.0
Cu-Sn(C)	120	−50	−2.14	40	60.2	0.0	1.2	38.6
Cu-Sn(C)	20	−100	−2.98	20	86.6	6.4	1.1	5.8
Cu-Sn(C)	40	−100	−2.95	27	64.2	22.9	3.3	9.6
Cu-Sn(C)	80	−100	−2.71	32	52.1	25.6	5.4	16.8
Cu-Sn(C)	120	−100	−2.54	37	64.8	0.0	0.0	35.2

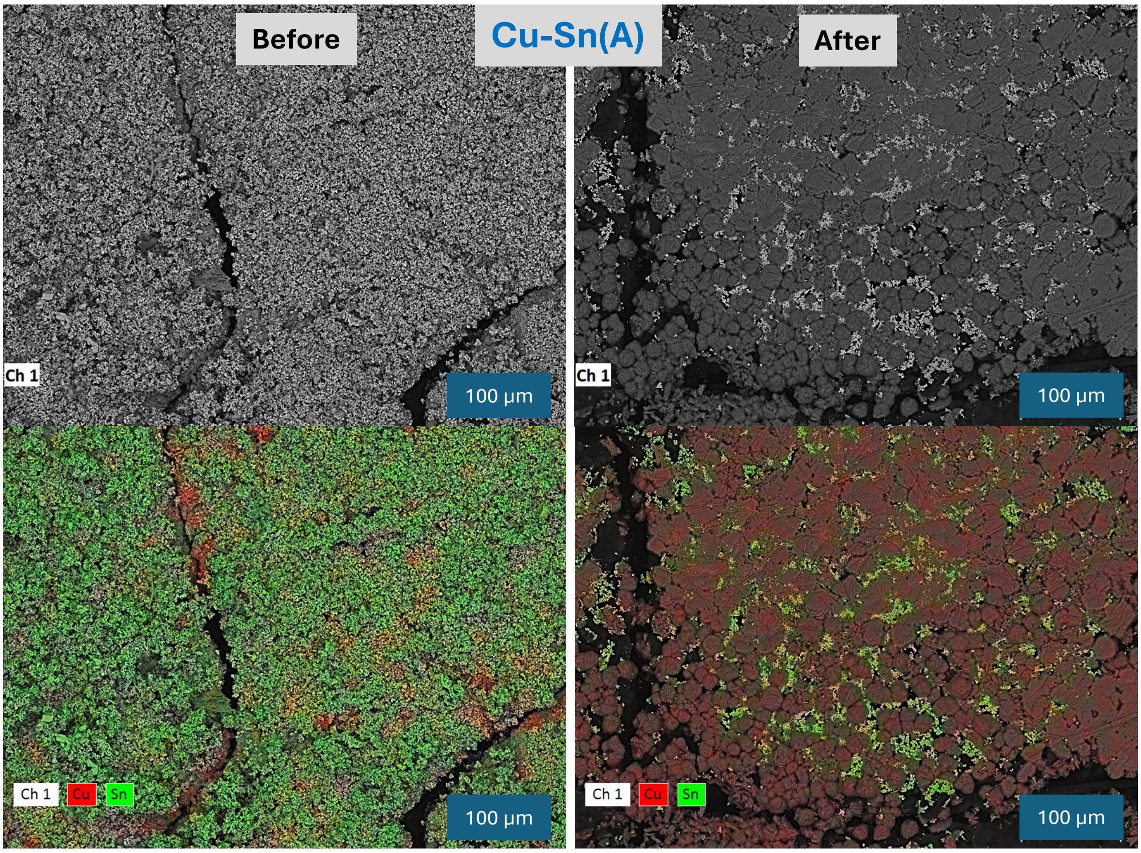

Figure S4. SEM/EDX mapping of catalyst Cu-Sn(A).

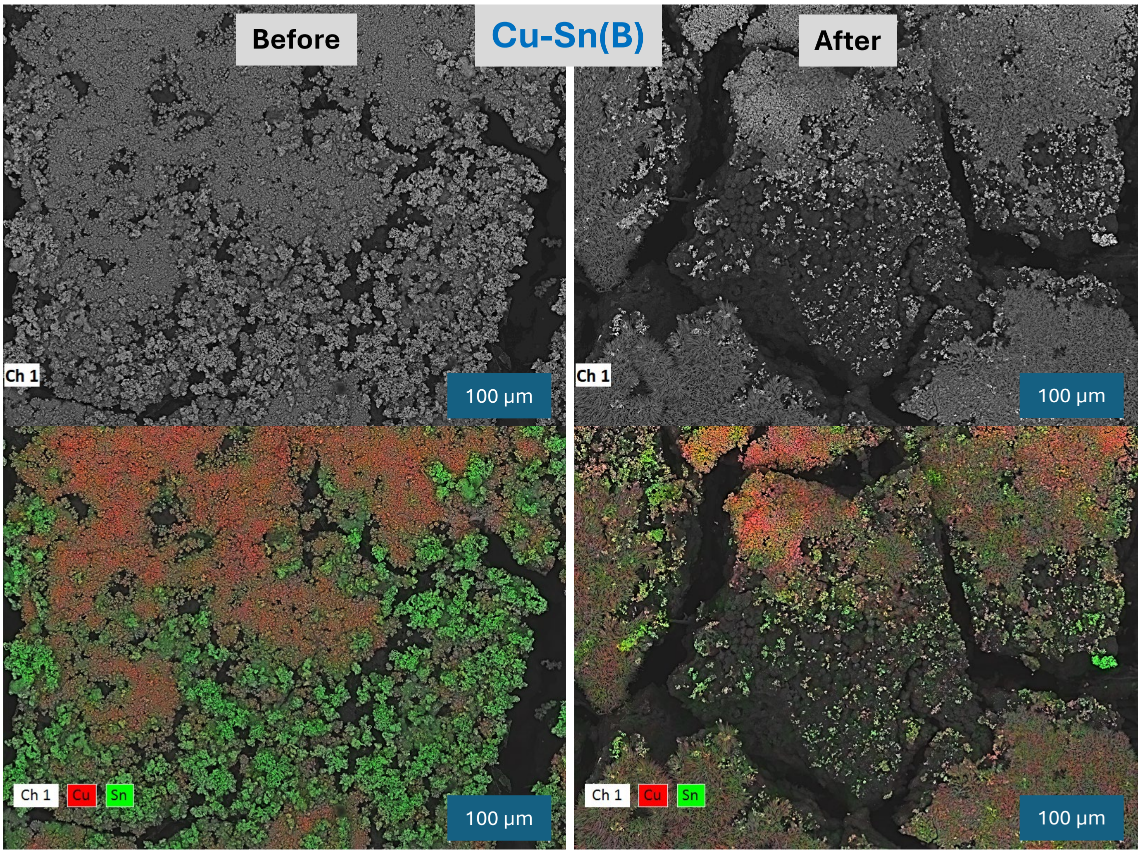

Figure S5. SEM/EDX mapping of catalyst Cu-Sn(B).

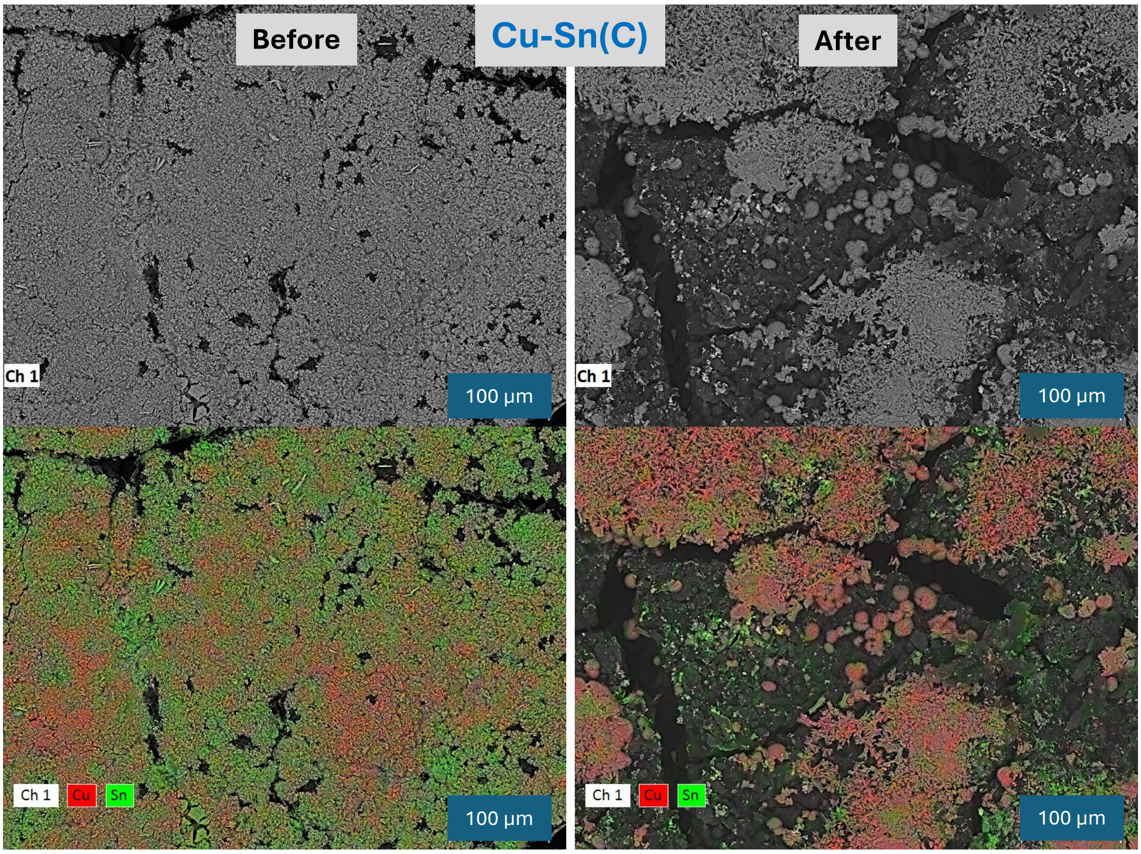

Figure S6. SEM/EDX mapping of catalyst Cu-Sn(C).
